# Supplementary material for: Impacts of clinical academic activity: qualitative interviews with healthcare managers and research-active nurses, midwives, allied health professionals and pharmacists
Source: BMJ Open. 2021 Oct 7;11(10):e050679. doi: 10.1136/bmjopen-2021-050679 (PMC8499282; doi:10.1136/bmjopen-2021-050679)
Supplement: Supplementary data [file bmjopen-2021-050679supp001.pdf]

Date ___ /___ /___ Interview code _____

Interview Topic Guide – Clinical Academic

Pre-interview

Consent form complete ☐
Check agree to audio recording ☐
Assure confidentiality ☐
Confirm time available for interview ____

Introduction

Aims of the whole service evaluation: explore clinical academic activity among non-medics at the Trust and how we can record the impact of this activity.
Aims of this interview are to find out about your experiences and any suggestions you have for this project.

Demographic information

Primary hospital site

☐ St Mary's Hospital
☐ Charing Cross Hospital
☐ Queen Charlotte's and Chelsea Hospital
☐ Hammersmith Hospital
☐ Western Eye Hospital
Other ____

NHS band ______________________________ **Year of clinical qualification** ____________________

Clinical discipline

☐ Nurse
☐ Midwife

☐ Clinical psychologists
☐ Healthcare scientists
☐ Pharmacists

☐ Assistant, technician or associate of any of these professions (also tick relevant professions)

☐ Other ______________________________
____

☐ Art, drama or music therapist
☐ Clinical research practitioner
☐ Dietitian
☐ Occupational therapist
☐ Orthoptist
☐ Operating department practitioner
☐ Osteopath
☐ Podiatrist
☐ Prosthetist/orthotist
☐ Paramedic
☐ Physiotherapist
☐ Radiographer
☐ Speech and language therapist

Clinical specialty ____

Research fellowships / funding ____
____
What does this fellowship/funding mean for you?

Newington L, *et al. BMJ Open* 2022; 11:e050679. doi: 10.1136/bmjopen-2021-050679

1. Can I start by asking: what your role involves day to day?
    Prompts
    Clinical v academic activities – are these integrated or separate?
    Is securing fellowship/research funding or part of role?
    Management
    Responsibility for supervising others
    Education
    Career aspirations
    What about after your fellowship?

2. What does the term clinical academic mean to you?
    Follow up:
    Do you see yourself as a clinical academic?
    If not, why not? And what might a clinical academic role look like to you
    What do you see as the difference between a clinical academic and an academic?

3. What do you think the role of clinical academics should be within:
    a) A local team, for example within your team
    b) The wider department
    c) Across the whole Trust
    Prompts
    What do you think are the benefits of these types of role?

4. How would you summarise your research activity at the Trust?
    Prompts
    Focus – clinical care/intervention, patient journey, experience
    How did you decide this was something you wanted to be involved with?
    Other clinical academic activities e.g. teaching / research support

5. Do you feel like your perspectives have changed as a result of your research involvement?
    Prompts
    Approach to delivering clinical care
    Approach to reviewing/appraising the evidence-base
    Has it changed how you feel about your work?
    Any change to how you see your career developing?
    Are there any activities you are now involved in as a result of your research/fellowship that might not have been possible otherwise?

6. What do you think are/have been/will be the impacts of your clinical academic activity?
    Prompts
    On patients – clinical care, pathways, experience, satisfaction
    On clinical team – have others become involved in research, clinical understanding, time management, project management, presentation skills, any cross over skill?
    To the Trust – staffing
    Outside the Trust – reputation, wider implication of change in practice

7. Are there any factors that were particularly helpful for you in generating these impacts?
    Prompts
    Protected time after fellowship
    Funding – publications, conferences etc

Newington L, *et al. BMJ Open* 2022; 11:e050679. doi: 10.1136/bmjopen-2021-050679

8. Have you experienced any obstacles to creating impact from your research?
    Prompts
    Protected time after fellowship
    Funding – publications, conferences etc
    Any differences between the opportunities for medical and non-medical HCPs

9. Where do you see yourself in 5-10 years time?
    Prompts
    Role – clinical/academic etc.
    Locations
    Goals

10. How do you think we can best capture and report the impact of our clinical academic activity?
    Prompts
    Discuss numerical versus qualitative/experiential data
    What type of data do you think hospital managers are looking for?
    What type of data do you think the NHS are/should be looking for nationally?
    Impact assessment tools/ standardised assessment measures
    Timescales of impacts – when to collect the impact data?

11. Which of these elements of impact do you think should be most important for the Trust?

Prioritisation activity using existing impact tool, plus anything else mentioned by interviewee above:
    Highlight the measures/questions you think are most important
    Prompt for top 3 measures of impact and reasoning for choices
    Are there any that you really don't think would be important for the Trust?
    How do you think this might be different for medics versus non-medics research?

12. Is there anything else you would like to discuss regarding clinical academic activity?

13. Do you have any questions you would like to ask me?

14. Is there anyone else in the Trust you think it would be particularly useful for me to speak to?

Post-interview

Thank interviewee for their time
Assure confidentiality – discuss interview/transcripts in batches
Inform re transcript check, if applicable

Newington L, *et al. BMJ Open* 2022; 11:e050679. doi: 10.1136/bmjopen-2021-050679

Date ___ /___ /___ Interview code _____

Interview Topic Guide – Manager

Pre-interview

Consent form complete ☐
Check agree to audio recording ☐
Assure confidentiality ☐
Confirm time available for interview ____

Introduction

Aims of the whole service evaluation: explore clinical academic activity among non-medics at the Trust and how we can record the impact of this activity.
Aims of interview: find out about your views and experiences as a manager of non-medical healthcare professionals

Demographic information

Primary hospital site

☐ St Mary's Hospital
☐ Charing Cross Hospital
☐ Queen Charlotte's and Chelsea Hospital
☐ Hammersmith Hospital
☐ Western Eye Hospital

Other ____

NHS band ______________________________

Clinical discipline (own background) ____

Discipline(s) of those responsible for:

☐ Nurse
☐ Midwife

☐ Clinical psychologists
☐ Healthcare scientists
☐ Pharmacists

☐ Assistant, technician or associate of any of these professions (also tick relevant professions)

☐ Other __________________________________
____

☐ Art, drama or music therapist
☐ Clinical research practitioner
☐ Dietitian
☐ Occupational therapist
☐ Orthoptist
☐ Operating department practitioner
☐ Osteopath
☐ Podiatrist
☐ Prosthetist/orthotist
☐ Paramedic
☐ Physiotherapist
☐ Radiographer
☐ Speech and language therapist

Clinical area /specialty ____

Newington L, *et al. BMJ Open* 2022; 11:e050679. doi: 10.1136/bmjopen-2021-050679

15. First, can I start by asking: what your role involves day to day?
    Prompts
    Any clinical duties
    Any involvement in research, service evaluation, audit
    Who are you responsible to – in terms of the management structure, rather than individual names

16. Have you received any funding or dedicated time for research as part of your career?
    If not, is this something you would have liked? – why?
    If yes, what did this mean to you?

17. What does the term clinical academic mean to you?
    Follow up:
    Do you have any individuals you would describe as clinical academics in your team?
    What about their role makes them a clinical academic?

18. Thinking generally, what do you think the role of clinical academics should be within their:
    d) Local team
    e) Wider department
    f) Trust

19. How do you think clinical academic roles might be supported within the NHS?
    Prompts
    Career structure
    Roles and responsibilities

20. What is your experience of managing team members involved in any type of clinical research activity?
    Prompts
    Types of research activity
    Managing backfill and recruitment
    Managing service delivery
    Role of the clinician within the team e.g. during a research fellowship
    Impact of the clinical academic activity on the wider team
    Any impacts to patient care
    Dissemination activities – writing for publication, presenting at conferences

21. What differences have these individuals made to your team?
    Prompts
    During the research
    After the research was finished // or what differences do you anticipate after the research is finished
    Impacts to patients
    Impacts to different team members
    Impact to the individual
    Positives and negatives

If no experience:
What do you anticipate might be the differences to your team if you were supporting a clinical academic within your service?
    Prompts

Newington L, *et al. BMJ Open* 2022; 11:e050679. doi: 10.1136/bmjopen-2021-050679

Backfill and recruitment
Service delivery
Role of the clinician within the team during research time
Impact of the clinical academic activity on the wider team
Any impacts to patient care
Dissemination activities – writing for publication, presenting at conferences
Differences both during and after their research project

22. Thinking broadly, what do you think are the impacts of clinical academic activity at the Trust?
    Prompt positives and negatives for the:
    Team
    Patients
    Individual
    Trust
    Professional discipline / other non-medical professions

23. How do you think we can best capture these impacts?
    Focus on interviewees ideas initially
    Prompt numerical data
    Prompt qualitative/experiential data – how do they think we could capture the value

Proceed to discuss contents of existing research impact frameworks/tools
Based around the VICTOR tool, which has 6 domains:
Health benefits, safety & quality improvements during the study
Service and work force impacts
Research profile of the organisation and research capacity
Economic impacts
Organisation's influence and reputation
Knowledge generation and knowledge exchange

24. What measures of impact do you think should be most important for the Trust?

Prioritisation activity using existing impact tool, plus anything else mentioned by interviewee above:
Prompt for top 3 measures of impact and reasoning
Are there any that you don't think would be important for the Trust?

25. Is there anything else you would like to discuss regarding clinical academic activity?

26. Do you have any questions you would like to ask me?

27. Is there anyone else in the Trust you think it would be particularly useful for me to speak to?

Post-interview

Thank interviewee for their time
Assure confidentiality – discuss interview/transcripts in batches
Inform re transcript check, if applicable

Newington L, *et al. BMJ Open* 2022; 11:e050679. doi: 10.1136/bmjopen-2021-050679
