# Supplementary material for: Impacts of clinical academic activity: qualitative interviews with healthcare managers and research-active nurses, midwives, allied health professionals and pharmacists
Source: BMJ Open. 2021 Oct 7;11(10):e050679. doi: 10.1136/bmjopen-2021-050679 (PMC8499282; doi:10.1136/bmjopen-2021-050679)
Supplement: Supplementary data [file bmjopen-2021-050679supp002.pdf]

Impact of non-medical clinical academics at Imperial College Healthcare NHS Trust (ICHT) – Information for participants

Thank you for your interest in our service evaluation exploring the impact of clinical academic activity among non-medical healthcare professionals. Key information about the project is outlined below. If you have any questions, or would like any additional information, please email Lisa Newington on l.newington@imperial.ac.uk.

Why are we carrying this service evaluation?

In 2018 a 5-year strategic plan was launched at ICHT to promote clinical academic activity among healthcare professionals outside medicine. The aims of the plan, entitled 'Research is Everyone's Business' were to increase research capacity and capability across these professional groups and to enhance the quality and relevance of research outputs to improve patient care. An additional aim was for ICHT to be seen as a leading NHS trust for clinical academic careers. This service evaluation has been developed to assess the perceived impact of clinical academic activity across non-medical healthcare professionals within ICHT. The findings will be used to develop a specific impact assessment framework that will enable the standardised capture of clinical academic research impact in the future.

Who is eligible to be involved?

We would like to speak to healthcare professionals of any grade who have been involved in clinical research at any level. This evaluation only includes non-medical professions, for example: nurses; midwives; allied health professionals (art therapists, dietitians, drama therapists, music therapists, occupational therapists, orthoptists, operating department practitioners, osteopaths, podiatrists, prosthetists/orthotists, paramedics, physiotherapists, radiographers, and speech and language therapists); clinical psychologists; healthcare scientists and pharmacists. Assistants, technicians and support workers within these disciplines are also invited to take part. We would also like to be speak to service managers for these professions.

Who is in the service evaluation team?

The evaluation is being led by Lisa Newington, with support from Caroline Alexander and Mary Wells. Contact details are provided at the end of this information sheet. A small number of research fellows and clinical students may also be involved in anonymised stages of the analysis, to provide an opportunity for their learning and development. Their invovlement will be supervised and moniored by the service evaluation team.

What will the interview involve?

The interview will be a one-off discussion with Lisa Newington. This will be guided by questions about your experience of being involved in clinical research, how you think clinical academic activity can be supported within the Trust and how we could/should measure the impact of this activity. The interview will be audio recorded to allow a qualitative analysis of the key themes across all interviews. If you would prefer to be interviewed with a colleague or colleagues, this can be accommodated. Interviews will be arranged at a time and location that is convenient for you and are anticipated to take 30-45 minutes. You will be given the opportunity to review the

interview text once it is transcribed, and to provide feedback on the initial analysis. It is up to you whether or not you wish to be involved in these steps.

Will my contribution be anonymous?

The recorded interview will be transcribed by an external company who are bound by a confidentiality agreement. Transcripts will be filed using an anonymous reference code. Identifiable data (your name and clinical specialty) will be logged separately. The project report for ICHT will include illustrative quotes to support the themes identified. It may be helpful to include participant names and clinical speciality in support of existing clinical activity, however these details will not be included without your expressed permission for the particular quote and in the specific context. Any journal publications that result from this project will not include any identifiable information.

Who has approved this project?

This service evaluation has been approved by the Imperial College Healthcare NHS Trust audit office (imperial.audit@nhs.net; reference 418). The project is funded by NIHR Imperial Biomedical Research Centre (BRC).

What if I no longer wish to be involved?

You can request for your interview to be removed from this project for 21 days after the interview, at which point the audio file and transcript will be deleted. After this time, the anonymous transcripts will have been incorporated into the analysis and it will not be possible to remove individual components. Before 01/08/2020, you can still request that no quotes from your interview are used in the final reports. Please email l.newington@imperial.ac.uk if you wish to make either of these requests.

Who should I contact if I have a complaint about the project?

Please speak to one of the service evaluation team in the first instant using the contact details below. Alternatively, you can contact the Trust audit office on 0203 312 2460 or imperial.audit@nhs.net.

Dr Lisa Newington
Research Associate
(Physiotherapist)
l.newington@imperial.ac.uk
07866997732

Dr Caroline Alexander
Lead Clinical Academic for Therapies, ICHT
caroline.alexander1@nhs.net
07884310240

Prof Mary Wells
Lead Nurse for Research, ICHT
mary.wells5@nhs.net
0203 311 7422

Thank you for your interest in our service evaluation!
